# Astrocytes and Aging

**DOI:** 10.3389/fnagi.2018.00337

**Published:** 2018-10-26

**Authors:** Alexandra L. Palmer, Shalina S. Ousman

**Affiliations:** ^1^Department of Neuroscience, Hotchkiss Brain Institute, University of Calgary, Calgary, AB, Canada; ^2^Departments of Clinical Neurosciences and Cell Biology & Anatomy, Hotchkiss Brain Institute, University of Calgary, Calgary, AB, Canada

**Keywords:** astrocytes, aging, inflammation, neurodegeneration, CNS, microglia, neurons, oligodendrocytes

## Abstract

By 2050, the aging population is predicted to expand by over 100%. Considering this rapid growth, and the additional strain it will place on healthcare resources because of age-related impairments, it is vital that researchers gain a deeper understanding of the cellular interactions that occur with normal aging. A variety of mammalian cell types have been shown to become compromised with age, each with a unique potential to contribute to disease formation in the aging body. Astrocytes represent the largest group of glial cells and are responsible for a variety of essential functions in the healthy central nervous system (CNS). Like other cell types, aging can cause a loss of normal function in astrocytes which reduces their ability to properly maintain a healthy CNS environment, negatively alters their interactions with neighboring cells, and contribute to the heightened inflammatory state characteristic of aging. The goal of this review article is to consolidate the knowledge and research to date regarding the role of astrocytes in aging. In specific, this review article will focus on the morphology and molecular profile of aged astrocytes, the consequence of astrocyte dysfunction on homeostatic functions during aging, and the role of astrocytes in age-related neurodegenerative diseases.

## Introduction

Aging is yet to be pinpointed to one specific factor or cell, but the current consensus is that aging is a multitude of factors (genetic, biological, environmental) that interplay together amongst the various cell types to contribute to physiological and cognitive changes characteristic of the aging process. Aging of the human brain is mainly observed as structural and physiological alterations that are most notably accompanied by cognitive decline. In terms of structure, imaging studies have shown that the aged brain displays a loss of dendritic spines (Pannese, 2011), decreases in oligodendrocyte (OL) number (Pelvig et al., 2008; Fabricius et al., 2013), stem cell loss in the hypothalamus (Zhang et al., 2017), and decreased brain volume across multiple neural regions (Ge et al., 2002; Resnick et al., 2003; Scahill et al., 2003; Raz et al., 2005; Freeman et al., 2008; Driscoll et al., 2009; Fjell et al., 2009; Li et al., 2013; Mu et al., 2017). The decrease in brain volume has been found to vary by sex, with males having more brain atrophy than females (Xu et al., 2000). Further, the reduced brain volume was hypothesized to be due to a loss of neurons (Brody, 1955; Coleman and Flood, 1987; Sturrock, 1989). However, a number of studies have shown that neuronal count does not change with age (Cragg, 1975; Terry et al., 1987; Freeman et al., 2008) and as such, the search for what was responsible (e.g., glia) for the structural changes became an active area of research.

With respect to physiological changes that occur with aging in the central nervous system (CNS), researchers observed an overall increase in gene expression in the brain (Berchtold et al., 2008; Boisvert et al., 2018). Although these genetic changes varied across brain regions and were sex dependent, with males showing more gene changes than females (Berchtold et al., 2008), there were some consistent changes. For example, across all brain regions, microglia and endothelial cells increased their gene expression levels, whereas astrocytes and OLs, but not neurons, shifted their expression pattern (Boisvert et al., 2018). Of note, these changes in gene expression in microglia, endothelial cells and astrocytes were of an inflammatory profile, which was intriguing given the well-known development of a mild inflammatory state with age (Howcroft et al., 2013; Morrisette-Thomas et al., 2014; Rea et al., 2018). Further, because the shift in gene expression pattern occurred in glial cells but not neurons, it implied that neurons are not a good indicator of age; instead, it has been proposed that glial specific genes may be a better predictor of age (Soreq et al., 2017). This review article will focus on what is known thus far about changes that occur to astrocytes during aging, the consequence of astrocyte dysfunction during aging on other resident CNS cells, and the role of astrocytes in age-related neurodegenerative diseases.

## Astrocytes

Astrocytes were first observed in the brain by Camillo Golgi in 1871. They are the largest cell population in the CNS with a ratio of astrocytes to neurons in the human cortex averaging 1.4:1 (Nedergaard et al., 2003). Astrocytes also represent the most heterogeneous group of glial cells and tile the entire CNS in a non-overlapping manner (Sofroniew and Vinters, 2010). Once considered to be no more than “brain glue” that provides structural support through extracellular matrix formation, astrocytes have now become known for key roles in a variety of complex and essential functions. A few of these functions include synapse development, neurotransmitter homeostasis, glycogen storage and blood brain barrier (BBB) maintenance (Sofroniew and Vinters, 2010). Due to their multi-faceted roles, it is not surprising that astrocytes are involved in aging.

### Phenotype and Molecular Profile of Aged Astrocytes

Astrocytes represent the most heterogeneous group of glial cells in terms of molecular, structural and physiological profiles and their classification relies on both morphological and molecular criteria. These glia are morphologically diverse, but are typically stellate-shaped cells with radial processes (Oberheim et al., 2009). With age in humans, there is an alteration in astrocyte phenotype from long and slender processes in young subjects to short and stubby processes in older individuals (Kanaan et al., 2010; Cerbai et al., 2012; Jyothi et al., 2015). This age-related morphological change has also been shown to occur in aged rodents (Castiglioni et al., 1991; Amenta et al., 1998) and primates (Kanaan et al., 2010; Robillard et al., 2016). With respect to numbers, there appears to be region-dependent differences in their density where reductions were noted in the retina of aged rodents (Mansour et al., 2008), while no changes were observed in the hippocampus (Lindsey et al., 1979; Jinno, 2011), and an increase seen in the human cortex (Hansen et al., 1987) and hypothalamus (Wang et al., 2006).

With respect to molecular criteria, glial fibrillary acidic protein (GFAP) has been used as a classical marker for astrocyte identification; although it is important to note that GFAP is not detectable in all astrocytes (Sofroniew and Vinters, 2010). Reports on aging astrocytes have shown that GFAP expression increases (Nichols et al., 1993; Kohama et al., 1995; Yoshida et al., 1996; Rozovsky et al., 1998; Wu et al., 2005; Clarke et al., 2018) and since an increase in GFAP expression is a common feature of reactive/activated astrocytes (Zamanian et al., 2012; Sofroniew, 2014; Liddelow et al., 2017), these findings suggest that astrocytes become reactive with age. More evidence in support of this notion was provided when it was found that astrocyte reactivity involves the differential expression of over 1,000 genes, some of which have been collectively attributed to “A1” reactive astrocytes—astrocytes of a more inflammatory state (Zamanian et al., 2012). Some of these “A1” reactive genes (Zamanian et al., 2012), such as complement system factors, antigen presentation molecules, secretory factors, peptidase inhibition and cholesterol synthesis, were recently identified as additional molecular profiles of aged astrocytes (Boisvert et al., 2018; Clarke et al., 2018). In addition, there is evidence that aged astrocytes undergo epigenetic changes and may alter BBB function and circadian rhythm.

### Aging Astrocytes and the Complement System

The complement system is part of the innate immune system, and aids in regulating inflammation as well as resistance to infection (Markiewski and Lambris, 2007). The system consists of approximately 30 soluble factors that are widely expressed in neurons and glia in the postnatal brain (Stevens et al., 2007; Hammad et al., 2018). C1q and C3 are major complement proteins that allow for cells to be tagged and targeted for phagocytosis (Stevens et al., 2007). During development, postnatal neurons express C1q at synapses which appears to target unwanted synapses for elimination (Stevens et al., 2007). In the mature brain, the complement system is dampened, however studies have shown that during CNS disease the system becomes enhanced by various cells, including astrocytes, and can lead to pathology via synapse, dendritic spine and neuronal loss, as well as changes in neuronal morphology (Stevens et al., 2007; Stephan et al., 2012; Zamanian et al., 2012; Lian et al., 2015, 2016). An increase in the genes belonging to components C3 and C4B is seen in astrocytes during aging (Boisvert et al., 2018; Clarke et al., 2018). C4B is a protein involved in both the classical and lectin pathway of the complement system. It is also a substrate that allows C3 convertase to cleave C3 into C3a and C3b, which direct both of these pathways toward opsonization and cell lysis. C3 is required for both these pathways, however it can also function on its own through the alternative pathway where it is involved in inflammation, cell migration, activation and cell lysis. This suggests that astrocytes are able to functionally partake in all three pathways of the complement system.

The activation of complement by aged astrocytes may contribute to cognitive decline. More specifically, because overall neuronal loss was not seen in aging humans, it has been suggested that changes in neuronal structure may instead be the culprit responsible for age-associated cognitive impairment (Berchtold et al., 2008). Since the current consensus is that memories are formed when neurons fire to one another, and recollection of that memory strengthens the associated synapses (Mayford et al., 2012), it is feasible that loss of a strengthened connection (synapse) between two neurons via complement targeting by reactive aged astrocytes could contribute to the memory deficits characteristic of elderly people (Choi et al., 2018).

### Antigen Presentation and Aging Astrocytes

The aging astrocyte also shows an upregulation in genes comprising major histocompatibility complex I (MHC I; Orre et al., 2014; Boisvert et al., 2018; Clarke et al., 2018), which is in agreement with other literature showing that there is an overall age-related increase in brain MHC I (Mangold et al., 2017). Interestingly, H2-K1 and H2-D1, both of which are upregulated in aging astrocytes and form part of MHC I, are associated with increased expression of pro-inflammatory genes (Mangold et al., 2017), implying that astrocytes could contribute to the low level inflammatory state that is characteristic of aging. That is, although contentious as to whether astrocytes present antigens *in vivo*, an increase in MHC I in astrocytes would suggest that these glia have an increased propensity to present antigens. More specifically, even though these glia are not professional antigen presenting cells (APCs) by definition, and they present antigens weaker than professional APCs, if only a subset of astrocytes presented antigens, the amount of overall antigen presentation would be large based on the sheer number of this glial cell in the CNS. Further support for the antigen presenting ability of aged astrocytes *in vivo* is their significant expression of various factors involved in phagocytic pathways, including *Pros1, Mfge8, Megf10, Lrp1* (Clarke et al., 2018); *Mertk* expression was not significantly increased, but was still expressed by aged astrocytes (Clarke et al., 2018). These results suggest that astrocytes possess the tools needed to tag (*Mfge8, Pros1, C3b*), phagocytose (*Mertk, MegF10, Lrp1*) and present (*H2-K1, H2-D1*) antigens. However, *Megf10, Mertk* and *Lrp1* expression does not necessarily signify negative consequences such as inflammation, because astrocytes may utilize these receptors for debris clearance. That is, astrocytes were found to possess lipid inclusions which contained myelin in the aging human optic nerve suggesting that astrocytes may be trying to clear myelin debris during aging (Nag and Wadhwa, 2012). Furthermore, apart from its immunological role, MHC I has been shown to be increased significantly with age in cognitively-intact individuals, but decreased in cognitively impaired individuals (Lazarczyk et al., 2016). This suggests that MHC I expression may have a positive role in brain aging by trying to preserve cognitive function. Thus, the antigen presentation capability of astrocytes with age may be both advantageous and detrimental depending upon the context. Further work needs to be done to elucidate exactly how these phagocytic factors in aged astrocytes are contributing to aging and whether they are detrimental, protective, or both.

### Aged Astrocytes and Secretory Molecules

Another characteristic feature of aged astrocytes is an increase in production of cytokines such as CXCL10/inducible protein-10 (IP-10; Clarke et al., 2018). CXCL10 serves as a chemoattractant for peripheral immune cells, and aids in T cell adhesion to endothelial cells (Sorensen et al., 2018). Interestingly, the receptor for CXCL10 is CXCR3, a microglia marker, suggesting that astrocytes and microglia may be communicating with each other during aging. This is entirely plausible as recent literature shows that microglia are able to induce reactivity in astrocytes (Liddelow et al., 2017; Rothhammer et al., 2018).

CXCL5 is another cytokine whose expression is augmented in aged cerebellar astrocytes (Boisvert et al., 2018). Like CXCL10, CXCL5 has chemotactic capabilities and has been shown to be involved in neutrophil recruitment^1^, and like astrocytes, neutrophils display aging deficits including an increase in their inflammatory properties (Adrover et al., 2016). Astrocytes have been shown to have both a direct (cell to cell) and indirect effect on polymorphonuclear neutrophils (Xie et al., 2010). That is, astrocytes are able to attenuate pro-inflammatory cytokine expression in neutrophils through direct contact, while an indirect interaction has been attributed to enhanced neutrophil necrosis (Xie et al., 2010). Therefore, it is plausible that aged astrocytes and aged neutrophils could synergistically elicit a heightened inflammatory response.

In addition to chemotactic and survival effects on immune cells via cytokine release, aged cortical astrocytes may negatively impact CNS health because of a decline in production of certain metabolic and trophic factors such as ATP and neurotrophins. For instance, a decline in ATP release may contribute to cognitive decline and impaired synaptic plasticity because ATP is involved in regulating neuronal activity through synaptic and tonic inhibition (Lalo et al., 2014). In addition, neuronal survival and neurogenesis may be decreased due to a reduction in the secretion of neurotrophins like vascular endothelial growth factor (VEGF), fibroblast growth factor 2 (FGF2; Bernal and Peterson, 2011) and brain derived neurotrophic factor (BDNF) in aged astrocytes (Bellaver et al., 2017).

### Oxidative Stress and Aging Astrocytes

Oxidative stress is a key component to aging and age-related diseases, including neurodegenerative diseases. The accumulation of oxidative damage, specifically to lipids, DNA, and proteins (Dizdaroglu et al., 2002; Cooke et al., 2003; Evans et al., 2004; Swain and Subba Rao, 2011), is proposed to result in age-associated functional losses in a process referred to as the free radical theory of aging or oxidative stress theory of aging (Beckman and Ames, 1998; Liguori et al., 2018). Indeed, increased reactive oxygen species (ROS) which cause damage to lipids, proteins, and DNA is frequently observed in aged tissue (Kudin et al., 2008; Brawek et al., 2010; Lukiw et al., 2012).

Middle-aged astrocytes have been shown to accumulate ROS and display an overload in Ca^2+^ (Ishii et al., 2017). Moreover, the over-abundance of Ca^2+^ was associated with an increase in JNK/SAPK activation, which is a member of the stress-activated MAPK signaling pathway, and which has been linked to cell death signaling (Ishii et al., 2017). Of further note, astrocytes that have been exposed to oxidative stress factors during aging begin to undergo oxidative stress themselves (Lei et al., 2008; Lu et al., 2008; Bellaver et al., 2017), and in age-associated neurodegenerative diseases such as multiple sclerosis (MS), astrocytes in active lesions have been shown to increase their accumulation of oxidized lipids and proteins in their cytoplasm (van Horssen et al., 2008) and oxidized DNA in their nuclei (Haider et al., 2011). Accumulation of oxidative stress suggests that these glia can no longer support neurons (Lin et al., 2007) which could lead to decreased neuronal function and damage as seen in the disease. However, whether this damaging mechanism really does occur in MS is unknown because astrocytes in active MS white matter (WM) lesions also augment expression of mitochondrial antioxidants (Nijland et al., 2014; Licht-Mayer et al., 2015; Lassmann and van Horssen, 2016). More specifically, enhanced expression of peroxisome proliferator-activated receptor-gamma coactivator 1-alpha (PGC-1α) was observed in reactive astrocytes, and astrocytes that over-express PGC-1α not only secrete less pro-inflammatory cytokines and chemokines, but they protect neuronal cells against oxidative attack greater than those co-cultured with control astrocytes (Nijland et al., 2014). Further, Nuclear factor erythroid 2-related factor 2 and its stabilizer and positive regulator, DJ1, is another antioxidant that is increased in reactive astrocytes in both active and chronic active MS lesions (van Horssen et al., 2010), and found to be protective against ROS in co-cultured neurons (Shih et al., 2003; Kraft et al., 2004; Calkins et al., 2005). Altogether, these findings imply that astrocytes are actively trying to combat oxidative damage both within themselves and neighboring cells, but their protective measures are insufficient to combat the overwhelming oxidative damage occurring with age and during age-associated neurodegenerative diseases.

### Aged Astrocytes and Peptidase Inhibition

Serine proteases control many key elements of the immune system including granzymes which activate apoptotic pathways, complement system proteins that mediate inflammation and phagocytosis, and production of cytokines and chemokines (Safavi and Rostami, 2012). These proteases have been linked to aging. For example, an increase in serine protease HTRA1 in mouse retinal pigment epithelium may contribute to age-related macular degeneration due to degeneration of the epithelium (Jones et al., 2011). Further, a significant increase in the serine proteases plasmin, trypsin and elastase was found in the blood of aged rats and appear to be involved in extracellular matrix degradation (Paczek et al., 2009). In aged astrocytes, serine protease inhibitor called Serpina3n is significantly upregulated (Boisvert et al., 2018; Clarke et al., 2018) which may be an attempt by aged astrocytes to combat the aging effects of serine proteases.

### Cholesterol Synthesis

Due to the BBB, the CNS does not uptake cholesterol from the blood stream, and instead has to locally synthesize the majority of cholesterol that it needs. Astrocytes are believed to play a major role in brain cholesterol synthesis because of their expression of sterol regulatory element-binding protein 2 (SREBP2; Ferris et al., 2017). SREBP2 activates the transcription of enzymes, including HMG-CoA reductase (*Hmgcr*), that are needed for cholesterol synthesis and cholesterol uptake receptors, including the LDL receptor (Madison, 2016). SREBP2 presence in astrocytes is critical for CNS function since its knockout in astrocytes in mice results in impaired brain development and function and reduced neurite outgrowth (Ferris et al., 2017). In the aged murine brain, the rate limiting cholesterol synthesis enzyme *Hmgcr* is downregulated, while the receptors for cholesterol transport are increased in astrocytes (Boisvert et al., 2018). This suggests that there is an overall dysregulation of the cholesterol synthesis pathway in astrocytes, and since many cells, including neurons, rely on cholesterol from astrocytes (Zhang et al., 2016), this dysregulation may lead to metabolic disruption in these nerve cells.

### Epigenetics of Aging Astrocytes

Studies that have looked at overall methylation changes during aging have shown that both hyper- and hypo-methylation occur with age at comparable rates (Maegawa et al., 2010; Issa, 2014). In addition, histone modification has also been shown to occur in aging cells (Wood et al., 2010; Liu et al., 2013). DNA methylation is an important process in the development of astrocytes since demethylation of astrocyte-specific genes such as GFAP, S100β and Aqp4 in neural stem cells (NSCs) promotes the switch from neurogenesis to astrogenesis (Namihira et al., 2004, 2009; Hatada et al., 2008; Takouda et al., 2017). In mature astrocytes, global DNA methylation patterns have been shown to occur in psychiatric disorders (Nagy et al., 2015) and alcohol use (Miguel-Hidalgo, 2018), however our understanding of epigenetic changes in astrocytes during aging is limited. Chrisholm and colleagues noted that H3K4 specific methyltransferase activity was lower in astrocytes from middle aged vs. young female rats following ischemia (Chisholm et al., 2015). As such, they proposed that future therapies may be able to target epigenetic modifications to provide neuroprotection against aging in astrocytes (Chisholm et al., 2015). Indeed, it has been suggested that inhibitors of histone deacetylases (HDACs) may be neuroprotective via effects on glial cells (Staszewski and Prinz, 2014). Some evidence in support of this presumption was seen in astrocyte cultures where it was shown that HDAC inhibition increased neurotrophic cytokines (Chen et al., 2006) and mitigated changes in Parkinson’s and Alzheimer’s disease (PD and AD; Nuutinen et al., 2010), although no changes were seen in reducing astrocyte activation (Xuan et al., 2012).

Environmental factors can also induce epigenetic changes. In a recent study that assessed how environment impacted young vs. old astrocytes, it was reported that aged mice from a standard environment could not distinguish between stationary and displaced objects (Diniz et al., 2016). Upon further examination of the astrocytes in these animals, the authors found two distinct morphological phenotypes and speculated that aging and environment reduce the complexity of astrocytes (Diniz et al., 2016). This agrees with previous literature showing morphological changes between young and old astrocytes (Kanaan et al., 2010; Cerbai et al., 2012; Jyothi et al., 2015). Furthermore, disturbances in astrocyte mitochondrial function have been shown to occur with age and these changes can also be seen when astrocytes are exposed to environmental neurotoxicants such as 3-chloropropanediol (Cavanagh et al., 1993), 1-methyl-4-phenyl-1,2,3,6-tetrahydropyridine (MPTP; Sundar Boyalla et al., 2011) and manganese (Zheng et al., 1998).

### Blood Brain Barrier (BBB) and Aged Astrocytes

The BBB consists of endothelial cells, astrocytic endfeet processes, pericytes, smooth muscle cells, neurons and perivascular microglia (Abbott and Friedman, 2012; Banks, 2016; Osipova et al., 2018). Considering that the BBB is comprised of such a variety of cell types, it is evident that disturbances in any one of these cells could result in changes to BBB integrity. The BBB is a compact complex held together by tight junctions which limits the diffusion of molecules and migration of cells from freely entering and exiting the brain. Transport of substances and cells that are required for CNS homeostasis occurs mainly by transcellular routes and acquisition of small molecules requires expression of specific transporters in order to pass across the BBB (Goodall et al., 2018).

As mentioned, astrocytes are an integral part of the BBB as they are involved in its development, maintenance and regulation. For example, astrocytes secrete Sonic Hedgehog (Shh) while BBB-endothelial cells bear the receptor for Shh (Alvarez et al., 2011). Blocking the Hedgehog pathway in astrocytes was found to increase barrier permeability while a lack of Shh led to compromisation of the barrier phenotype (Alvarez et al., 2011). Astrocytes also secrete a variety of factors that influence the function and permeability of the BBB. For instance, inflammatory factors such as nitric oxide, IL-6, TNF-α and matrix metalloproteinases, which astrocytes are known to secrete when they acquire an inflammatory state, have been shown to impair vasculature control and function (Zhang, 2008). A common pathway that is involved in the production of these inflammatory factors includes the NF-κB pathway, which has been shown to have a direct effect on the tight junction proteins occludin, ZO-1 and claudin-5 (Lamberti et al., 2001; Stamatovic et al., 2008; Aveleira et al., 2010). It is also important to note however, that astrocytes can also be protective in BBB function. This includes the NF-κB pathway, which has been shown to promote barrier function by inducing the expression of Shh (Kasperczyk et al., 2009).

BBB changes have been documented in neurodegenerative diseases, which are typically associated with age progression (Kirk et al., 2003; Zlokovic, 2008). However, changes in BBB permeability with age are conflicting as some groups have shown an increase in permeability with age (Mooradian and McCuskey, 1992; Hosokawa and Ueno, 1999; Kirk et al., 2003; Hafezi-Moghadam et al., 2007; Farrall and Wardlaw, 2009; Popescu et al., 2009; Goodall et al., 2018) while others have shown no alteration (Wadhwani et al., 1991; Vorbrodt and Dobrogowska, 1994; Banks et al., 2000; Mackic et al., 2002). Due to this dichotomy, further research needs to be done to clarify the changes that are occurring with age, and specifically how the aged astrocyte contributes to these changes. Based on the changes that we know occur in aging astrocytes we can speculate that if there is age-dependent BBB alterations, aged astrocytes could contribute to any phenotype and permeability impairment because of their inflammatory secretions.

#### Chronodisruption and Aged Astrocytes

An interesting observation about aged astrocytes is their association with chronodisruption. Circadian rhythm generates a time clock to a 24 h cycle that influences our behavior and physiology and adapts us to light/dark cycles of the earth (Kondratova and Kondratov, 2012; Hood and Amir, 2017). Our circadian rhythm is generated in the suprachiasmatic nucleus (SCN) of the hypothalamus via a transcriptional/translational feedback loop (Golombek and Rosenstein, 2010; Duhart et al., 2013). Briefly, the loop begins when transcription factors, CLOCK and BMAL1 heterodimerize and initiate transcription of *Per* and *Cry* genes. PER and CRY proteins form a complex in the cytoplasm and block transcription of CLOCK/BMAL1, which in turn inhibits transcription of *Per* and *Cry*. PER/CRY complex is also targeted for degradation via the proteasomal pathway, which initiates the transcription of CLOCK/BMAL1 again. This feedback loop forms the core oscillator mechanism (Lowrey and Takahashi, 2011; Duhart et al., 2013). The SCN is composed of a variety of classes of neurons and astrocytes (Abrahamson and Moore, 2001). Recent work has demonstrated that SCN neurons are metabolically active during daytime while SCN astrocytes are active during night and that these astrocytes are able to suppress the activity of SCN neurons through extracellular glutamate (Brancaccio et al., 2017).

Disruption of circadian rhythm has been suggested to be carcinogenic to humans (Straif et al., 2007) and has been well documented to be altered during aging and neurodegenerative diseases of aging (Kondratova and Kondratov, 2012; Mattis and Sehgal, 2016). Oxidative stress has not only been proposed as a mechanism of aging, and is indeed a common feature amongst aging neurodegenerative diseases, but it is speculated to contribute to changes in circadian rhythm with age (Grimm et al., 2011; Kondratova and Kondratov, 2012; Krishnan et al., 2012). As discussed above, oxidative stress negatively impacts astrocytes by reducing their ability to support neurons. Because the neuron-astrocyte relationship is integral in the SCN, disruption of proteins in the SCN could perturb this collaboration. Indeed and for example, BMAL1 deletion in SCN neurons can cause partial astrocyte activation, while BMAL1 deletion in SCN astrocytes induced astrogliosis and astrocyte dysfunction (Lananna et al., 2018). In addition, circadian disruption can induce GFAP expression (Lananna et al., 2018), while aging (Wyse and Coogan, 2010) and inflammation (Curtis et al., 2015) have been shown to suppress BMAL1 levels, thus potentially influencing astrocyte activation state as well (Lananna et al., 2018). However, loss of BMAL1 in astrocytes does not disrupt circadian rhythm (Tso et al., 2017). Instead, it was found that deletion of CK1ε, an enzyme that controls phosphorylation of PER/CRY in SCN astrocytes, resulted in an increase in behavioral period in mice (Tso et al., 2017). These results suggest a feedback loop involving astrocytes and neurons in circadian rhythm impairment. That is, the reciprocal relationship between astrocyte and neurons in the SCN suggests that a disruption in astrocytes would in turn affect neurons resulting in disruption of the feedback loop.

#### Summary

**Figure 1:**
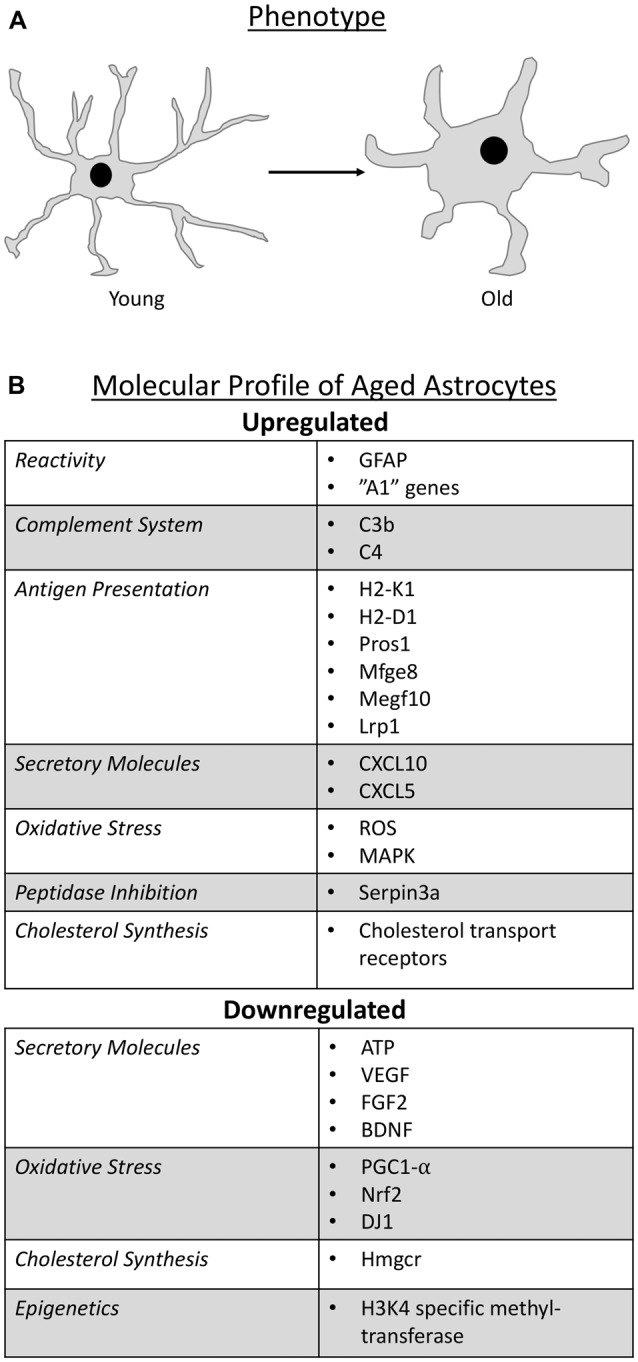
Schematic representation that summarize the phenotype change of astrocytes as they age **(A)** and the molecular profile of aged astrocytes **(B)**.

The majority of changes in aged astrocytes may be fueling the pro-inflammatory processes that occur with aging—albeit there are instances (e.g., serine protease inhibition) where aged astrocytes are trying to combat detrimental mechanisms (Figure 1). Since the majority of these alterations are of the pro-inflammatory nature, it appears that the anti-inflammatory processes within astrocytes and other CNS cells are inadequate to quell the inflammatory properties, thus contributing to a heightened inflammatory state as we age. It is likely also that the enhanced expression of pro-inflammatory changes in aged astrocytes has effects beyond these glia by influencing the function of other CNS cells, the subject of the next section.

## Aged Astrocytes and Other CNS Cells

### Microglia

It was a long-held dogma that glial cells only provided support to neurons, however recent research suggests a much more complex and active interplay between glial cells themselves and their relationship with neurons. While more work is needed to definitively elucidate the relationship between different glial cells during aging, there are some insights into what is occurring amongst these cells during senescence. In a model of aging neuroinflammation in the hippocampus, astrocyte branches were found to be actively bisecting the cell body of neurons while microglia contained neuronal debris (Cerbai et al., 2012), suggesting that astrocytes and microglia may both be involved in eliminating neurons during aging. Whether these are joint efforts or each cell performing their function autonomously is however unclear. Recent work from the Barres lab demonstrated that activated microglia are able to influence astrocyte activation through their secretory profile (e.g., C1q, TNF-α and IL-1α; Liddelow et al., 2017) implying that microglia could influence astrocyte activation during inflammation. In the context of aging, microglia have been shown to increase their reactivity (Hickman et al., 2013; Norden and Godbout, 2013; Grabert et al., 2016), which would suggest that they would augment their secretory profile. There is some support for this idea since it has been shown that the increase in reactive astrocyte genes associated with aging was significantly reduced in mice lacking C1q, TNF-α and IL-1α (Clarke et al., 2018). Of no surprise, this feedback loop between microglia and astrocytes is also present when trying to resolve inflammation, of which becomes disrupted in the aging brain. More specifically, activated microglia secrete the anti-inflammatory cytokine IL-10. Astrocytes express the IL-10 receptor (IL-10R) and upon activation of this receptor, secrete TGF-β, which in turn decreases microglial activation (Norden et al., 2014). However, aged astrocytes do not increase their expression of IL-10R or secretion of TGF-β, which would release the brakes needed to suppress astrocyte and microglial activation during aging (Norden et al., 2014). Therefore, astrocytes and microglia function both autonomously and non-autonomously in aging and age-related neurodegenerative disorders to contribute to neuronal loss and inflammaging of the brain (Franceschi et al., 2007).

### Neurons

Astrocytes support neuronal function in many ways including ion homeostasis, metabolic support and transmitter homeostasis (Sofroniew and Vinters, 2010). As mentioned previously, neuronal numbers do not change with age (Cragg, 1975; Terry et al., 1987; Freeman et al., 2008), but their function and morphology change with age (Pannese, 2011). This led researchers to explore whether age-related changes in astrocytes could impact neuronal function. Indeed, astrocytes gain an “A1” phenotype with age, and previous work with this phenotype found that “A1” astrocytes secrete a neurotoxin that kills neurons (Liddelow et al., 2017). In addition, aging astrocytes increase expression of *Sparc* (Boisvert et al., 2018), which blocks synapse formation and decreases α-amino-3-hydroxy-5-methyl-4-isoxazolepropionic acid receptors (Nobuta et al., 2012; Allen, 2014) thereby potentially inhibiting synapse function of neurons. As mentioned previously also, upregulation of the complement system factors, C3 and C4B in aged astrocytes, suggests that these glia have the potential to eliminate neuronal synapses (Boisvert et al., 2018; Clarke et al., 2018). Finally, disruption in lipid metabolism (see “Cholesterol Synthesis” section) could lead to impaired synapse formation and function of neurons (van Deijk et al., 2017). However, it should be noted that the majority of astrocyte homeostatic functions involved in neuronal support are unaltered with age, including genes involved in ion, glutamate and lactate regulation (Boisvert et al., 2018). This suggests that astrocytes are still capable of supporting certain neuronal functions with age and thus the balance between the various beneficial and detrimental effects will determine the health of a neuron. For instance, although the majority of astrocyte support of neuronal cells is not altered, the changes that occur in every aged cell (neuron, microglia, OL) that influence neuronal function may eventually add up and tip the balance towards an impaired neuron.

### Oligodendrocytes

In regard to OLs, there is some evidence that astrocyte dysfunction with age could negatively impact the function of these cells. OLs synthesize myelin and wrap axons that are seen as WM in the brain and spinal cord. Aging results in a 30% decrease in WM and a 45% decrease in myelinated fiber length from the age of 20–80 years old (Bartzokis et al., 2001; Marner et al., 2003). While myelin production still occurs as we age, aging results in thinner myelin sheaths and shorter internodes (Marner et al., 2003) that can be traced back to OL dysfunction. The hypothesis is that due to oxidative stress, OLs suffer from DNA damage that accumulates during aging to result in WM changes (Tse and Herrup, 2017). In addition to OLs, precursors for these glia may also contribute to the WM changes with age. In the mature CNS, a pool of progenitor cells known as OL progenitor cells (OPCs) reside to serve as replacements for OLs. While the OPC pool remains constant throughout age (Boda et al., 2015) there is an inherent defect in their differentiation with senescence. Their differentiation time is doubled (Zhu et al., 2011) and there is an impairment in their recruitment to replace OLs (Doucette et al., 2010).

Astrocytes are known to support OL function including proliferation, differentiation and myelination. While OLs synthesize cholesterol to produce myelin they also rely on cholesterol synthesis from astrocytes for their myelin production; however, as discussed above, cholesterol synthesis is impaired in aging astrocytes. As a consequence, OLs may not receive the necessary amount of cholesterol during aging, thus resulting in reduced myelin production characteristic of the aging brain. Another process of OLs that aged astrocytes may disrupt is differentiation. Before OLs can produce myelin they need to undergo differentiation from their OPC state. FGF2, an important factor in OPC differentiation (Bögler et al., 1990), is decreased in aged astrocytes (Bernal and Peterson, 2011), suggesting that astrocytes may hinder the differentiation process.

In contrast to the detrimental effects aging astrocytes may have on OLs and OPCs, they may also be potentially beneficial. Astrocytes increase their expression of CXCL10, which is involved in the migration of OPCs (Omari et al., 2005), and an increase in the inflammatory state of aging astrocytes coincides with an increase in STAT3 (Monteiro de Castro et al., 2015), that may be in involved in crosstalk with microglia and OPCs to prevent impairment of OPC maturation (Nobuta et al., 2012). It is possible however, while these mechanisms are integral in recruiting OPCs to sites that need OL repopulation and differentiation, the astrocyte-contributing defects in differentiation and reduced myelin production may overcome these beneficial properties of aged astrocytes on OL functions.

### Summary

**Figure 2:**
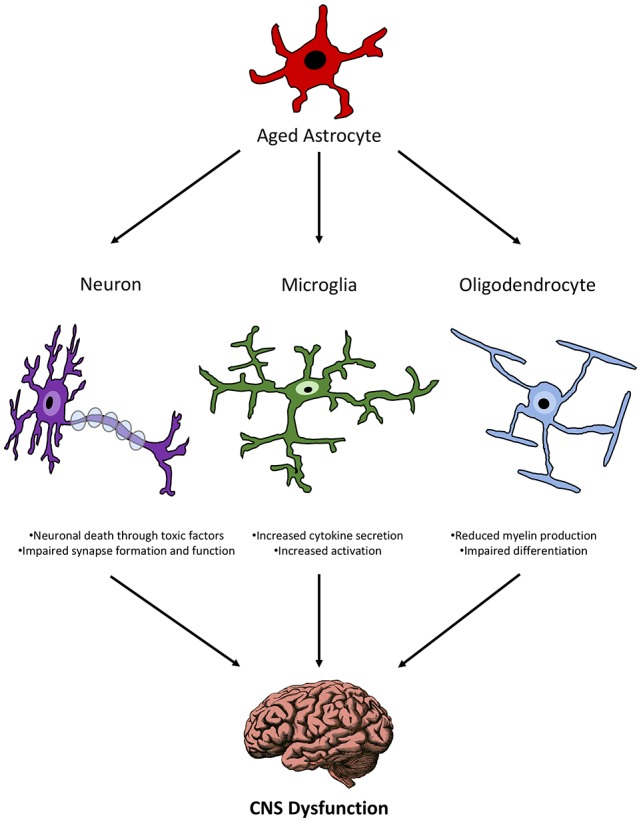
Schematic representation of the relationship between aged astrocytes and other CNS cells during senescence.

The interactions of aged astrocytes with other CNS cell populations suggest that they can propagate inflammation and directly affect the health and function of these other cells (Figure 2). Since all of these cells communicate with each other, a direct effect of astrocyte interactions with any one of these cell populations can create a feedback loop that results in the dysfunction of multiple cell types and ultimately functional impairment as seen in age-related diseases.

## Aged Astrocytes and Neurodegenerative Diseases

In 2015, the aged population (aged 65 and over) represented 8.5% (or 617.1 million) of the total population (He et al., 2016). By 2050, it is predicted that the aging population will rise to over 16.7% or 1.6 billion (He et al., 2016). That is a 150% expansion of the aged population (He et al., 2016). With age comes an increased risk for age-related diseases, which includes neurodegenerative diseases. Aging and neurodegeneration share many features including gliosis, loss of myelination, cognitive decline, loss of working memory and astrocyte dysfunction. AD, PD and amyotrophic lateral sclerosis (ALS) are three common age-related diseases known to have astrocyte involvement. There are many in-depth reviews on AD, PD and ALS and thus we will limit our discussion to what is known about the role of astrocytes in these diseases.

### AD and Astrocytes

AD affects more than 35 million people worldwide and is the most frequent form of dementia in the aged population, accounting for 50%–56% of cases (Querfurth and LaFerla, 2010). Aging does not mean individuals will progress to AD, however, AD and aging share many common features including oxidative stress, mitochondrial dysfunction, inflammation, proteotoxicity and altered gene expression (Chakrabarti and Mohanakumar, 2016). These common features can also be found in AD astrocytes. For example, neurons in AD have been shown to increase insulin-like growth factor 1 (IGF-1) via astrocyte interactions, which can lead to increased beta-amyloid formation by neurons (Costantini et al., 2010). Beta-amyloid, in turn, is capable of stimulating astrocytes and causing activation of NF-κB pathways (considered an important mediating agent in neuroinflammation in AD; Akama and Van Eldik, 2000; Wang et al., 2013; Shi et al., 2016) and consequently production of pro-inflammatory cytokines such as IL-1β, IL-6, iNOS and TNF-α (Bales et al., 1998; Akama and Van Eldik, 2000; Hou et al., 2011). Other evidence of astrocyte involvement in AD pathogenesis is a decline in antioxidant defense, which could result in the accumulation of oxidative damage in astrocytes leading to neurodegeneration (Hung et al., 2010). For example, oxidative stress can result in activation of astrocytes (Andersen, 2004) and activation of NADPH oxidase in astrocytes, which has been shown to lead to beta-amyloid-induced neuronal death through mitochondrial dysfunction (Abramov et al., 2004).

### Astrocytes and PD

PD is the second most common age-related disease after AD (Reeve et al., 2014). Age is the strongest risk factor for PD, with prevalence of the disease increasing more than 400 times as one ages (Rodriguez et al., 2015). Although volume reduction can be seen throughout the aging brain, the substantia nigra dopaminergic neurons are particularly affected in PD displaying neuronal loss at a rate of 4.7% to 9.8% per decade (Fearnley and Lees, 1991; Ma et al., 1999; Rudow et al., 2008). It is incompletely known why dopaminergic neurons are targeted, but it has been suggested that astrocytes may be involved. Astrocytes and neurons share a very close relationship due to the fact that astrocytes are part of the tripartite synapse, provide structural and metabolic support, buffer neurotransmitters, and help regulate synaptic transmission (Sofroniew and Vinters, 2010). Because these functions become compromised in the aged astrocyte, it is likely that neuronal health would be directly impacted. Furthermore, genes that have been identified as part of astrocyte biology are also causative in the development of PD; these include *PARK7, SNCA, PLA2G6, ATP13A2, LRRK2, GBA, PINK1* and *PARK2* (Zhang et al., 2016; Booth et al., 2017). These genes are involved in a variety of functions in astrocytes including inflammatory responses, cholesterol synthesis, and mitochondrial dysfunction which are also known to be dysfunctional in PD.

### ALS and Astrocytes

ALS is a degenerative disease of motor neurons in the brain, brain stem and spinal cord, that leads to paralysis and death (Rowland and Shneider, 2001). Although ALS is not considered a prototypical aging disease, the median age of onset occurs at 70.8 years of age in European populations (Marin et al., 2014) and has an incidence curve similar to that of PD (Logroscino et al., 2015). In addition, recent genetic advances have revealed that pathways involved in the development of the disease are also modulated during the aging process. These include autophagy, inflammation and cellular maintenance (Niccoli et al., 2017). The precise mechanisms that target motor neurons in ALS remain elusive. However, it has been proposed that during aging in rodents a loss of astrocytic support to motor neurons may result in loss of both motor neurons and their function (Das and Svendsen, 2015; Das et al., 2016). This loss of support is further accelerated in a rodent model of ALS (Das and Svendsen, 2015). Furthermore, astrocytes generated from post-mortem brain tissue from both familial and sporadic ALS are toxic to motor neurons and a knockdown of the superoxide dismutase 1 gene in astrocytes, a known genetic risk factor in ALS, attenuates this toxicity (Haidet-Phillips et al., 2011). In addition, treatment of motor neuron/astrocyte co-cultures with an inhibitor for necrotic cell death prevented loss of motor neurons (Re et al., 2014). It appears that the NF-κB signaling pathway in astrocytes may be participating in the astrocyte-motor neuron death (Haidet-Phillips et al., 2011).

### Summary

The general inflammatory state of astrocytes gives us a good prediction of different processes that may become dysfunctional during disease. By exploring how this inflammatory state is manipulated or altered in various age-related neurodegenerative disorders, we can gain better insight into potential disease mechanisms and pathology.

## Conclusion

In conclusion, a growing number of studies have shown that astrocytes play a more central role than previously appreciated in aging and age-related diseases. These glia become more pro-inflammatory and contribute to the general low-grade inflammation that is characteristic of the aging brain. Further, because of their active interactions and secretory products, aged astrocytes could negatively afflict other CNS cells. Further work is needed to unravel how these glia interact with other CNS cells at the cellular and molecular level since this will have implications when developing therapeutics for age-related diseases.

## Author Contributions

SO and AP conceived and wrote the manuscript.

## Conflict of Interest Statement

The authors declare that the research was conducted in the absence of any commercial or financial relationships that could be construed as a potential conflict of interest.

## Footnotes

^1^https://www.ncbi.nlm.nih.gov/gene/6374
